# Disability and Depression Improve after Lumbar Discectomy, But May Not Change for Other Causes of Back Pain

**DOI:** 10.5812/kowsar.22287523.1996

**Published:** 2011-09-26

**Authors:** Scott D. Daffner

**Affiliations:** 1 Department of Orthopaedics, West Virginia University School of Medicine, West Virginia, USA

**Keywords:** Low back pain, Depression, Discectomy

*Dear Editor*,

The recent article by Farzanegan et al. on the effects of lumbar discectomy on the rates of disability and depression among patients with chronic low back pain is interesting (1). Their study suggests that among patients with more than 3 months of back pain due to a herniated disc, surgical treatment (particularly laminectomy-discectomy) can improve both disability and depression. Their paper highlights 2 important points: first, chronic pain conditions can lead to increased disability and depression, and, second, successful treatment of these conditions can improve the patients’ disability and depression.

Their data indicate that an improvement in disability and depression is not observed in all patients. Interestingly, the number of patients scored as moderately, severely, or very severely depressed (14, 7, and 1, respectively) was the same at 6 and 12 months after the operation. This finding suggests that a certain cohort of patients might have a preexisting baseline depression at the onset of disc herniation, and that the depression is not caused by their chronic pain condition. The association between chronic low back pain and depression has been well described previously. Recently, Reme et al. reported that the incidence of psychiatric disorders among a population with chronic low back pain was 31% with a 4% prevalence of major depression (2). Many patients have somatization disorders and back pain is frequently a presenting condition of depression. Thus, it is important to recognize this group of patients because their symptoms may not improve with surgical intervention.

Furthermore, the authors only report on a distinct subgroup of patients with chronic low back pain-those with associated disc herniations. These patients have an excellent clinical outcome after surgical intervention and report some of the highest patient satisfaction ratings of all spinal surgeries (3). Surgical treatment of “discogenic” low back pain, however is much less predictable and results in generally poorer outcomes; in fact, outcomes for lumbar procedures vary depending on the underlying diagnosis (4, 5). Therefore, these results cannot be generalized to the entire population of patients with chronic low back pain.

Although I commend the authors on reporting these important results from their study, these results must be viewed within the context of the study and may not be widely applicable. While surgical intervention may improve disability and depression among patients with lumbar disc herniation, it may not improve these factors in patients with significant underlying preexisting psychological conditions. Moreover, further research is required to determine the effects of surgery on depression and disability for other pathological conditions associated with chronic back pain.
